# Royal Australian and New Zealand College of Psychiatrists professional practice guidelines for the administration of repetitive transcranial magnetic stimulation

**DOI:** 10.1177/00048674241249846

**Published:** 2024-05-05

**Authors:** Salam Hussain, Suneel Chamoli, Paul Fitzgerald, Ashu Gandhi, Shane Gill, Shanthi Sarma, Colleen Loo

**Affiliations:** 1 Division of Psychiatry, Faculty of Health and Medical Sciences, The University of Western Australia, Perth, WA, Australia; 2 Consultation Liaison Psychiatry and Neuromodulation, Sir Charles Gairdner Hospital Mental Health Service, Perth, WA, Australia; 3 Binational Committee, Section of Electroconvulsive Therapy and Neurostimulation, The Royal Australian & New Zealand College of Psychiatrists, Melbourne, VIC, Australia; 4 TMS Specialists Clinics, Neuropsytech Pty Ltd, Canberra, ACT, Australia; 5 School of Medicine and Psychology, Australian National University, Canberra, ACT, Australia; 6 Department of Psychiatry, Monash Health, Melbourne, VIC, Australia; 7 Rehabilitation, Mental Health and Chronic Pain Clinical Institute, Epworth Clinic, Melbourne, VIC, Australia; 8 Discipline of Psychiatry, School of Medicine, The University of Adelaide, Adelaide, SA, Australia; 9 South Australian Psychiatry Training Committee, The Royal Australian & New Zealand College of Psychiatrists, Adelaide, SA, Australia; 10 The Adelaide Clinic, Ramsay Mental Health Care, Adelaide, SA, Australia; 11 Mental Health and Specialist Services, Gold Coast Hospital and Health Service, Gold Coast, QLD, Australia; 12 Medicine Department, Faculty of Health Sciences & Medicine, Bond University, Gold Coast, QLD, Australia; 13 School of Psychiatry, University of New South Wales, Sydney, NSW, Australia; 14 The Black Dog Institute, Randwick, NSW, Australia; 15 The George Institute for Global Health, Barangaroo, NSW, Australia

**Keywords:** Guidelines, repetitive transcranial magnetic stimulation, neurostimulation, psychiatric disorders, professional practice, neuromodulation, depression

## Abstract

**Objectives:**

To provide guidance for the optimal administration of repetitive transcranial magnetic stimulation, based on scientific evidence and supplemented by expert clinical consensus.

**Methods:**

Articles and information were sourced from existing guidelines and published literature. The findings were then formulated into consensus-based recommendations and guidance by the authors. The guidelines were subjected to rigorous successive consultation within the RANZCP, involving the Section of ECT and Neurostimulation (SEN) Committee, its broader membership and expert committees.

**Results:**

The RANZCP professional practice guidelines (PPG) for the administration of rTMS provide up-to-date advice regarding the use of rTMS in clinical practice. The guidelines are intended for use by psychiatrists and non-psychiatrists engaged in the administration of rTMS to facilitate best practice to optimise outcomes for patients. The guidelines strive to find the appropriate balance between promoting best evidence-based practice and acknowledging that evidence for rTMS use is a continually evolving.

**Conclusion:**

The guidelines provide up-to-date advice for psychiatrists and non-psychiatrists to promote optimal standards of rTMS practice.

## Introduction

Repetitive Transcranial Magnetic Stimulation (rTMS) is a form of localised brain stimulation therapy. It involves the focal application of a localised, pulsed magnetic field to the cerebral cortex, inducing small electrical currents which stimulate nerve cells in regions of the brain implicated in regulation of mood and other psychiatric symptoms. The precise mechanism of action is not yet fully understood. Studies suggest that activation of nerve cells in targeted brain regions can change local regional brain activity and connection of relevant, brain networks (Anderson et al., 2016). rTMS is a non-invasive form of therapy that does not require sedation with anaesthesia.

The purpose of these guidelines is to provide information for psychiatrists and non-psychiatrists who are using rTMS as a treatment modality. These guidelines are not intended as a directive about clinical practice, or instructions as to what must be done for a given patient.^1^ It is important to recognise that the circumstances of each individual can vary. As a consequence, each patient’s assessment to undergo rTMS and their rTMS treatment plan may differ. Consideration should be given to each patient’s presentation, so treatment is individually and clinically tailored.

rTMS for depression is funded under the Medicare Benefits Schedule (MBS) in Australia. While these guidelines provide advice on appropriate clinical practice, they do not provide advice on billing practices or interpretation of MBS item numbers. Further information about rTMS on the MBS is available on the Department of Health and Aged Care website.

## Methodology

To develop the guidelines information was sourced from existing international guidelines and published literature focusing on peer-reviewed empirical studies, particularly meta-analyses and systematic reviews, peer-reviewed case studies/reports and standards of care documents. These guidelines are not designed to be a full academic review of rTMS. A formal literature search methodology was not used. Literature was sourced regarding specific issues as discussed by the RANZCP Bi-national Section of Electroconvulsive Therapy and Neurostimulation (SEN), and findings formulated into consensus-based recommendations and guidance. The guidelines are a significant update of the RANZCP rTMS guidelines published in 2018, with information provided for an increased range of psychiatric conditions, patient selection, rTMS treatment variables, maintenance rTMS, retreatment with rTMS, and skills and training. The guidelines were subjected to rigorous successive consultation and review within the RANZCP involving over 400 members of the SEN in August 2023, College committees, and expert and clinical advisors with an interest in rTMS.

Throughout this process, the draft guidelines were regularly reviewed by the SEN Executive Committee at its bi-monthly meeting. The guidelines were approved in line with RANZCP processes by the Practice, Policy and Partnerships Committee, the Corporate Governance and Risk Committee, and finally by the RANZCP Board in December 2023.

## When can rTMS be used?

In clinical practice rTMS should only be administered for an illness where there is adequate evidence of clinical indication and effectiveness. It should be considered as a therapeutic option alongside other treatments after detailed psychiatric assessment. A summary of evidence and appropriate use in various psychiatric conditions is listed below. Guidance on appropriate clinical practice for these conditions is listed in the section on ‘rTMS administration’.

A substantial and growing evidence base for the use of rTMS in several disorders comes from double-blind, randomised controlled trials using sham rTMS as a control comparator, as well as open label studies and reports of outcomes in large real-world clinics. In the sham-controlled studies, considerable care was taken to ensure all the experience of rTMS treatment, including attendance at multiple treatment sessions, engagement with treaters, the process of arranging the treatment coil on the head, was equivalent for the sham/placebo treatment group, with the only difference being the lack of active magnetic stimulation.

Although evidence for rTMS is strongest in depression, and to a lesser extent schizophrenia, there is research being undertaken in the benefits of rTMS for a wide range of other conditions that psychiatrists may encounter across a range of areas of practice including treatment of addiction, chronic pain and eating disorders. While evidence is still emerging, it is helpful for all psychiatrists and non-psychiatrists to understand the potential role for rTMS in these situations.

### Depression

The primary clinical indication for rTMS is in the treatment of depression. rTMS is suitable for treatment of a major depressive episode that has not responded to, or is unsuitable for, adequate treatment with medications and, where appropriate, psychotherapeutic approaches.

The development and clinical implementation of rTMS for depression have been supported by a robust evidence base. This includes an extensive series of double-blind, randomised, sham-controlled clinical trials which demonstrate the efficacy of rTMS and numerous meta-analyses and network meta-analyses (Berlim et al., 2013; Brunoni et al., 2017; Mutz et al., 2018; Papadimitropoulou et al., 2017; Sehatzadeh et al., 2019). Evidence supporting the efficacy of rTMS can be found in a recent umbrella review of meta-analyses of randomised controlled trials investigating the effectiveness of all forms of non-invasive brain stimulation for depression (Razza et al., 2020) and internationally published consensus recommendations (McClintock et al., 2017). Data on real-world effectiveness in the United States reported response rates of 58–83% for rTMS treatment of depression (Sackeim et al., 2020).

Some efficacy trials included subsets of patients with bipolar depression, demonstrating similar antidepressant response rates as patients experiencing unipolar depression, although few studies have targeted this group in stand-alone trials (Mutz et al., 2018). The efficacy of TMS in treating psychotic depression is unclear. For patients with depression that is very severe, associated with psychotic features, highly treatment resistant, or requires a rapid response due to acute risk, clinicians will need to consider whether treatment with Electroconvulsive Therapy (ECT) is more appropriate (Brunoni et al., 2017).

There is also evidence supporting rTMS to treat anxious depression and anxiety symptoms that are comorbid in depression (Chen et al., 2019; Clarke et al., 2019). There is no convincing evidence that any rTMS protocol, including specific site or frequency, is superior when it comes to anxiolytic effects. It is worth mentioning that Medial Prefrontal Cortex (MPFC) was a focus of one multicentre study that suggested higher efficacy in anxiety, anhedonia and psychomotor retardation types of depression (Drysdale et al., 2017).

### Schizophrenia

The evidence base for the use of rTMS in schizophrenia is less substantive than that for depression (Galletly et al., 2016). Clinical trials have examined the use of rTMS to treat symptoms of schizophrenia, particularly auditory hallucinations, negative symptoms and cognitive deficits.

Meta-analysis and review of these studies concluded there is evidence of efficacy for rTMS with a low frequency, inhibitory stimulus applied to the left temporoparietal region as a treatment for auditory hallucinations (Matheson et al., 2010; Otani et al., 2015; Slotema et al., 2014) and that these benefits may persist over clinically valuable periods of time (Hoffman et al., 2003). The majority, but not all, randomised controlled trials involving a sham control comparison, have found rTMS applied to the auditory cortex to have beneficial effects in reducing the severity and/or frequency of auditory hallucinations (Kennedy et al., 2018). Compared to sham, rTMS improved hallucinations and negative symptoms but was associated with modest, non-significant worsening of positive symptoms (Kennedy et al., 2018).

Currently there is no clear evidence for the use of rTMS in improving cognitive impairments including language decline in people with schizophrenia, highlighting the need for more multicentre randomised controlled trials in the field (Hasan et al., 2016; Shishkovskaia et al., 2022).

A relatively large number of studies now have tested whether rTMS, usually applied to the left dorsolateral prefrontal cortex (DLPFC), can have therapeutic benefits in patients experiencing negative symptoms. A review in 2022 identified 57 studies with a total of 2633 patients and included the results from all of the studies in a meta-analysis (Lorentzen et al., 2022). The analysis showed clear superiority of active over sham stimulation and a number needed to treat of 5. The major issue with this literature is the lack of studies demonstrating longer term benefits (Garg et al., 2016; Tseng et al., 2022).

Given the lack of therapeutic options for patients who have persistent auditory hallucinations or negative symptoms, despite optimal medication treatment, it would seem reasonable to offer rTMS therapy in centres with specialist training and where data on outcomes are collected for quality assurance and analysis. While rTMS as add-on therapy to standard care for the treatment of refractory hallucinations in schizophrenia may not meet standard cost-effective thresholds compared to standard care alone. Given the refractory nature of this condition and the relatively small size of this population, it may be reasonable for decision-makers to adopt a higher incremental cost-effectiveness ratio threshold (Hendriks et al., 2022).

### Obsessive–compulsive disorder (OCD)

There is accumulating evidence supporting the use of rTMS in OCD with recent meta-analyses reporting positive findings (Grassi et al., 2023; Perera et al., 2021; Rehn et al., 2018; Zhou et al., 2017). However, a wide variety of treatment targets and paradigms used in studies to date and pooling of paradigms in some meta-analyses make it difficult to draw firm conclusions.

In 2018 the FDA in the US-approved marketing of a Deep TMS device for the treatment of OCD based on a trial of 100 medication resistant patients. This device uses a ‘deep TMS’ coil, that is, a technology that is different to standard rTMS, and rTMS was combined with an activation paradigm. Additional ‘real-world’ studies have provided evidence for efficacy (Roth et al., 2021) and this approach appears cost-effective (Gregory et al., 2022). Deep rTMS can also be delivered using an angled figure of eights coil linked to a standard TMS device – some devices have been Therapeutic Goods Administration (TGA)-approved for treatment of OCD in Australia.

Given the considerable limitations of current treatment approaches for OCD, the use of deep TMS in patients failing to respond to standard treatment approaches is justifiable. Further evidence is required to define the methods of application of standard rTMS before this can be recommended for routine clinical use.

### Post-traumatic stress disorder (PTSD)

There is accumulating evidence supporting the use of rTMS in PTSD. It may be a promising alternative or add-on treatment for PTSD patients who show limited response to antidepressant medication and/or trauma-focused psychotherapy (Kan et al., 2020). A recent meta-analysis of TMS studies in PTSD identified 11 randomised controlled trials. Promisingly, the authors found that TMS produced a significant reduction in core PTSD symptoms with a large effect and these benefits lasted at least four weeks after treatment finished (Kan et al., 2020). However, beneficial effects in PTSD have been seen with both high- and low-frequency stimulation, and stimulation applied to both the left and right DLPFC, and as such the optimal treatment paradigm is not yet clear.

### Anxiety

Recent meta-analyses have identified rTMS as a promising treatment for generalised anxiety disorder (GAD) (Cirillo et al., 2019; Parikh et al., 2022). A meta-analysis including six studies involving a total of 152 patients (97 patients who received active treatment and 55 who received placebo) found that, collectively, the results suggested that rTMS treatment for GAD reduced anxiety scores across all populations, suggesting that rTMS has promise as a treatment for adults with GAD (Parikh et al., 2022). Furthermore, TMS was associated with improved emotion regulation in GAD (Parikh et al., 2022). This indicates that rTMS may have significant clinical utility in patients with GAD, if these results can be replicated in satisfactory clinical studies. However, there are few randomised sham-controlled clinical trials, and further research is warranted (Parikh et al., 2022).

### Eating disorders

Application of multi-session rTMS to eating disorders has yielded promising but as yet inconclusive results, both in relation to bulimia nervosa and binge eating disorder. Findings in the context of anorexia nervosa are more controversial, with evidence of improvement in affective functioning, but a trend of iatrogenic weight loss (Hall et al., 2018).

For anorexia nervosa, only one sham-controlled study with repeated sessions performed in a sample size larger than 10 patients has been published to date. In this study, 32 patients with anorexia nervosa lasting for at least 3 years were equally randomised to receive 20 sessions of either real or sham HF-rTMS to the left DLPFC. The real stimulation was superior to sham rTMS especially for mood outcomes, rather than for eating disorder symptoms or weight gain (Lefaucheur et al., 2020).

No beneficial effects were detected for primary outcomes (i.e. BMI, binge eating and compensatory behaviours; urge to binge and to eat; severity of EDs symptoms) among individuals with AN, BN and other EDs-NOS. rTMS showed moderate therapeutic effects on affective functioning (i.e. negative affectivity, depressive and anxious symptoms) of individuals with EDs (Cavicchioli et al., 2022). For multi-session treatment of clinical conditions, more studies are needed for rTMS (Hall et al., 2018). Thus, it is still premature to consider rTMS therapy for eating disorders in clinical practice (Lefaucheur et al., 2020).

### Addiction

The therapeutic potential of rTMS on addiction, by targeting craving in particular, has been explored with heterogeneous results (Zhang et al., 2019). A meta-analysis using updated evidence assessed overall rTMS efficacy on craving, differential effects between addiction types clustered into three groups: depressant (alcohol, cannabis, opiate), stimulant (nicotine, cocaine, methamphetamine) and behavioural addiction (gambling, eating disorder). Analyses performed using random effects models revealed a small effect size favouring active rTMS over sham TMS stimulation in the reduction of craving. It found a significant difference between addiction types, with a persistent small effect only for stimulant and behavioural addiction groups. There was no difference between the different combinations of target and frequency of stimulation, but a significant correlation between number of sessions and craving reduction. Recommendations on optimal stimulation settings and its clinical application await further research (Gay et al., 2022).

rTMS has also been investigated as a potential augmentation strategy for current treatments for opioid use disorder with promising results although further research is needed before strong conclusions can be drawn (Young et al., 2020).

rTMS has been identified as a safe and promising therapeutic technique for the management of comorbid schizophrenia and substance use disorders, with the majority of evidence in tobacco use disorder. Larger trials are needed establish the efficacy of rTMS in reducing drug consumption and craving in psychotic patients, ideally in comparison to existing pharmacological and behavioural intervention (Johnstone et al., 2022).

There is some evidence for the use of rTMS in smoking cessation (Gay et al., 2022). Studies are also ongoing into the use of rTMS on sustained tobacco abstinence, a meta-analysis of seven sham-controlled studies were included (*n* = 699 patients) with promising results suggesting that rTMS may improve smoking abstinence rates from 3 to 6 months after quitting smoking, compared with sham or usual treatment (Petit et al., 2022).

Some devices have been TGA-approved for treatment of psychoactive substance use disorders (PSUD) caused by drugs with stimulatory effect on brain function in adult patients in Australia. Deep TMS has been approved for clinical use by the FDA in the United States for smoking cessation.

### Chronic pain

A large number of studies have explored the use of TMS in the treatment of chronic pain although these have varied substantially in the pain syndrome targeted and the type of TMS used. Benefits have been seen in studies applying stimulation to the primary motor cortex (M1), to the DLPFC and using deep TMS. The most consistent promising effects seen in clinical trials have been present when stimulation has been applied to the left M1 although it has been proposed that optimal treatment should involve a systematic evaluation of multiple treatment targets in an individual patient (Lefaucheur and Nguyen, 2019).

A meta-analysis conducted to characterise the potential analgesic effects of high-frequency rTMS over the DLPFC on chronic pain identified no overall effect of TMS across chronic pain conditions, although there was a significant short-term analgesia in neuropathic pain conditions only (Che et al., 2021). Furthermore, significant analgesic effects in chronic pain were demonstrated with stimulation of the M1 site although optimal protocols have not yet been confirmed (Lefaucheur and Nguyen, 2019).

### Post-stroke depression and cognition

A meta-analysis evaluating the efficacy of rTMS in patients with post-stroke depression (PSD) specifically comparing rTMS with control condition for PSD defined mean change in depression symptom scores as the primary efficacy outcome (Shao et al., 2021). Secondary outcomes included the remission rate of depression, stroke recovery and cognitive function recovery. In total, 7 randomised control trials with 351 participants were included. At post-treatment, rTMS was significantly more effective than the control condition. However, no significant difference was found for cognitive function recovery between the two groups. This highlights that rTMS could be an effective treatment for patients with PSD (Shao et al., 2021). Further clinical studies with larger sample sizes and clearer subgroup definitions are needed to confirm these outcomes (Kim et al., 2020; Shao et al., 2021).

### Other

rTMS has also been investigated for use in a range of other disorders such as autism spectrum disorder and ADHD. A variety of these other potential uses have been explored in clinical trials of varying number and size, with data summarised by Lefaucheur et al. (2020). The preliminary therapeutic evidence in these other disorders varies but in no area have large scale multi-site trials or meta-analyses to date established efficacy. There are multi-site trials underway in several other conditions and it is likely that the range of approved applications for rTMS could develop rapidly.

At present there is insufficient evidence for the clinical use of modified forms of rTMS, for example the Magnetic e-Resonance Therapy (MERT) approach, in the treatment of any mental disorder, including autism spectrum disorder and ADHD.

## Patient selection

The screening and selection of patients appropriate for rTMS treatment is essential and should be conducted by a psychiatrist. All psychiatrists undertaking assessment and prescription of rTMS should be adequately trained and have a detailed understanding of when rTMS is clinically indicated and contra-indicated.

Sufficient information and time should be provided to patients considering rTMS before consent is sought. Psychiatrists should consider and discuss the risks and benefits of rTMS with the patient before recommending a treatment course. Psychiatrists should inform patients of costs that will be associated with a course of rTMS treatment, given it is most commonly available in private practice. Patient-focused information on rTMS is available and should be referred to as a source of information for the community.

Valid consent is essential for patients considering rTMS and should be sought in line with Principle 5 of the RANZCP Code of Ethics. The consent process must be undertaken by a psychiatrist with knowledge and expertise in rTMS therapy. Details should be provided about the treatment methodology, process, possible adverse events and what to expect before, during and after the administration of the treatment. During the consent process, psychiatrists should ensure patients understand that therapeutic outcomes of rTMS cannot be guaranteed.

It would be appropriate for families, carers and whānau to be involved in the consent process, depending on the patient’s preference. The provision of rTMS to a patient lacking capacity to provide informed consent should only occur with appropriate substituted approval/consent as per local regulations. rTMS is not a regulated treatment under any mental health act. As with any other treatment, psychiatrists should be aware of ethical and practical implications if treating patients involuntarily with rTMS.

When psychiatrists are considering rTMS treatment for their patients but do not have detailed knowledge of rTMS, it is recommended that the psychiatrist seek advice from a psychiatrist with current and appropriate rTMS experience to determine a patient’s suitability to undergo rTMS.

The risk/benefit ratio should be carefully considered before recommending a treatment course in certain groups:

**Pregnant women:** There are no large, standardised trials assessing safety data on the use of rTMS in pregnant women and current evidence is limited to case series. The theoretical risk of rTMS is thought to be low due to the rapidly dissipating magnetic field from the stimulation coil and the distance of the foetus from the coil (Taylor et al., 2018) and available case reports have not found that rTMS given to a mother during pregnancy has any detrimental effects on the foetus (Pridmore et al., 2021). Nonetheless, the use of rTMS in this group requires a careful assessment of the patient’s situation and detailed informed consent. Careful discussion of known and potential risks of rTMS compared to alternative modalities of treatment is warranted. It is advisable for these discussions to include other family members as appropriate. Outcomes in this group should be closely monitored and where possible, reported to inform a growing empirical evidence base.
**Children and adolescents:** There is limited safety and efficacy data on the use of rTMS in children and adolescents, although recent reviews suggest that rTMS may be a promising treatment option for adolescents with depression (Hett et al., 2021; Sigrist et al., 2022). rTMS should only be given to those aged under 18 within an approved research protocol or under circumstances where there are limited other treatment options and potential clinical benefit is considered to outweigh the risks of treatment in this group. This would require a careful assessment of the patient’s circumstances, family situation, developmental stage and maturity, capacity to provide informed consent, including an understanding of known and potential risks. Consent should be obtained in line with section 5.8 of the RANZCP Code of Ethics and the opinion of the child and adolescent’s treating psychiatrist should be sought. Outcomes in this group should be closely monitored and where possible reported to inform an empirical evidence base. A service using a specific TMS device should check the intended use has been formally approved by the TGA in Australia or in line with requirements of the New Zealand Medicines and Medical Devices Safety Authority, as the age group specifications can differ between devices. Where there is no approved listing for the device for the age group and the disorder, the patient and primary care giver must be advised of its off-label use.
**Older people:** Although rTMS studies specifically in older populations are scarce, there is no evidence that rTMS is less effective in older patients, and there are no additional safety concerns (Gálvez et al., 2015; Sackeim et al., 2020).
**Pre-existing conditions:** While the overall incidence of seizures induced by rTMS is very low, attention needs to be directed to the assessment of seizure risk factors, such as pre-existing neurological conditions, past seizures, comorbid alcohol/substance use and changes in concurrent medications during the rTMS course, some of which may reduce seizure threshold (Taylor et al., 2018).
**Hearing protection:** During stimulation sessions, rTMS produces a loud clicking noise which can cause discomfort and affect hearing. Hearing protection, such as the use of ear plugs, is therefore advised. rTMS can present a problem for those with metal implants or electronic devices, for example cochlear implants or pacemakers and its use in this population with a safe distance between the rTMS coil and the metal/electronic device considered (Taylor et al., 2018).

## rTMS administration

### Clinical settings for rTMS

rTMS can be delivered in a hospital inpatient or outpatient setting. It can also be delivered in non-hospital outpatient settings, such as a medical clinic. Worldwide, the majority of rTMS is conducted on an outpatient basis. rTMS treatment does not require sedation or general anaesthesia.

All services providing rTMS should have in place appropriate protocols, training and equipment to allow for the safe and effective administration of treatment. This should include protocols for patient assessment, monitoring during treatment, monitoring of the quality of the provision of treatment, protocols for response to adverse events and monitoring of outcomes.

Where rTMS is conducted as an outpatient the outpatient rTMS clinic should be suitably accredited by an accepted accreditation agency such as International Standards Organisation (ISO) or Australian Council of Healthcare Standards (ACHS).

Devices used for rTMS should be approved by the TGA for use in Australia or in line with requirements of the New Zealand Medicines and Medical Devices Safety Authority for use in New Zealand. A service using a specific TMS device should check the intended use, as these can differ between devices.

The treatment of non-approved conditions or those without substantial research evidence should only be undertaken as part of research or clinical trials.

### rTMS treatment variables

There is a wide range of variables which can be modified in the delivery of rTMS (e.g. stimulation coil, stimulation site, stimulation frequency, stimulation intensity, frequency of treatment sessions, number of stimulation pulses or trains applied per session and total duration of rTMS course). These are determined by the treating psychiatrist as part of the prescription procedure, depending on the condition of the patient. All psychiatrists prescribing rTMS should have undertaken training and have sufficient expertise to allow for the appropriate choice of rTMS stimulation parameters.

In clinical practice, rTMS should follow protocols derived from (and proven effective by) substantive clinical trials. If rTMS is prescribed in a manner that deviates from the standard stimulation parameters derived from clinical trials, patients receiving the treatment should be informed and the reasons for this clearly documented. rTMS services consistently using non-standard stimulation protocols should only do this within a research protocol approved by the local research ethics committee. This includes significant variations in rTMS scheduling, stimulation frequency, intensity and site.

Table 1 gives a guide for treatment options. It is noted that this is a rapidly evolving field so readers should continue to keep updated with current literature to inform best practice regarding clinical indications and treatment advances. There is not sufficient systematic randomised data comparing all forms of rTMS used in clinical practice to confidently recommend commencing with one over another. Therefore, commencing with left high frequency (LHF), right unilateral low frequency (RLF), left intermittent theta burst stimulation (iTBS) and sequential bilateral are all supported by the evidence that is available.

**Table 1.** Treatment option protocols for rTMS.

Depression
Established protocols
The intensity, or magnetic field strength, of rTMS is usually set as a percentage of the patient’s motor threshold (MT), defined as the minimum stimulus strength required to induce a small involuntary muscle contraction (usually in the thumb of the contralateral upper limb) assessed visually or with the aid of electromyography. The majority of large multi-site trials have provided stimulation at 120% of the Resting Motor Threshold (RMT) but earlier trials also showed efficacy and lower intensities (90–110% of the RMT), and a recent study showed TBS to be efficacious at 80% (Chen et al., 2021). rTMS coil placement in the treatment of depression is usually determined using one of the three approaches: (1) A modified 6 cm rule (research has shown that the earlier 5 cm approach is inadequate in targeting the appropriate region in the prefrontal cortex and is not recommended), (2) Targeting based on the 10–20 EEG system (e.g. at the F3/F4 EEG electrode, such as the F3 Beam method) or (3) Neuronavigation technique(s) using 3D MRI imaging (Fitzgerald, 2021). Most rTMS clinical trials have evaluated treatment applied as single sessions on a daily basis, 5 days per week for between 4 and 6 weeks but the largest multi-site trials have provided treatment for up to 6 weeks and several have also included a tapering schedule following this (O’Reardon et al., 2007). This has been the standard treatment schedule in routine clinical applications using established protocols.
**High-frequency stimulation applied to the left dorsolateral prefrontal cortex (DLPFC).** This is the form of rTMS treatment for which there is the largest evidence base and is the most commonly used. The evidence base includes over 30 independent clinical trials including two large multi-site studies (Brunoni et al., 2017; George et al., 2010; O’Reardon et al., 2007; Razza et al., 2020). The FDA approval of rTMS in 2008 was based on a trial with stimulation applied to the left DLPFC at an intensity of 120% of the patient’s resting motor threshold (RMT), total of 3000 pulses for each session, lasting 37.5 minutes (high frequency, 10 pulses/second in a 4 second train with a 26 second pause between trains). The FDA has approved the protocol delivered with a shorter inter-train interval (11 seconds) (Jin et al., 2023).
**Low-frequency stimulation applied to the right DLPFC.** This form of treatment uses continuous 1 Hz trains. It has been evaluated in more than 10 clinical trials and there are multiple studies demonstrating therapeutic equivalence between low-frequency right-sided rTMS and high-frequency left-sided rTMS (Berlow et al., 2020). Right-sided treatment is usually applied in a single 20- to 30-minute train to the right DLPFC at 120% of the RMT. This approach is usually better tolerated than high-frequency stimulation (Kaur et al., 2019). It is associated with lower seizure risk and is routinely used in patients who have struggled with side effects to left-sided treatment.
**Sequential bilateral rTMS** This involves a combination of the two approaches described above applied at each stimulation session. The majority of research suggests that sequential bilateral TMS is more effective than sham but no more effective than unilateral stimulation protocols (Li et al., 2022; Mutz et al., 2019).
Other protocols
**Deep rTMS** This refers to stimulation that is applied with a proprietary ‘H-coil’ produced by one rTMS device manufacturer. Deep rTMS is usually applied at a high frequency to the left DLPFC but with a deeper and less focussed magnetic field. A systematic review and meta-analysis found that, when matched on frequency, the higher-intensity and less focal stimulation with the H1-coil reduces depression more than the lower-intensity and more focal stimulation but further research is needed identify optimal stimulation protocols for acute and longer-lasting efficacy (Gellersen and Kedzior, 2019). At this stage Deep TMS has demonstrated at least equivalent efficacy and is a reasonable alternative to standard left sided rTMS applied with a figure of eight coil (Filipčić et al., 2019; Levkovitz et al., 2015).
**Theta burst stimulation (TBS)** Theta burst stimulation (TBS) is a form of rTMS that has shown similar effects to standard rTMS with significant advantage in administration time (Blumberger et al., 2018). TBS is increasingly applied to treat depression (Chen et al., 2021). TBS’s brevity and evidence base is well-suited to application in accelerated schedules. A single large non inferiority trial found that the daily application of a single 3 minute intermittent TBS (iTBS) session applied to the left DLPFC produced equivalent effects to standard rTMS leading to approval of this protocol by the FDA in the US and increasingly common use in standard clinical practice (Blumberger et al., 2018). Additional clinical trials have verified the clinical efficacy and safety of iTBS for depression (Clinical TMS Society, 2023).
**Other accelerated treatment protocols** Research studies have explored ‘accelerated’ treatment approaches. Several studies have indicated that accelerated forms of rTMS and TBS can achieve similar clinical outcomes requiring fewer treatment sessions (Chen et al., 2021; Fitzgerald et al., 2018). One accelerated approach using a very high dose schedule and MRI based targeting has demonstrated a rapid onset of clinical benefits and high overall response rate (Cole et al., 2022). However, this schedule requires 10 treatments per day over 10 hours and is likely to be challenging to implement in clinical practice. Further research is continuing to define the optimal accelerated protocols.
**Auditory Hallucinations for Schizophrenia**
**Low frequency stimulus to left auditory cortex** This approach involves the daily application of low frequency (1 Hz) stimulation to the left temporoparietal cortex (TP3 EEG point) with the coil orientation as originally described by Hoffman et al. (Brunelin et al., 2023; Hoffman et al., 2003; Slotema et al., 2014)
**Negative symptoms of schizophrenia**
**High-frequency forms of rTMS applied to the left DLPFC** A number of studies, supported by meta-analysis (Lorentzen et al., 2022; Tseng et al., 2022), have indicated that excitatory/high-frequency forms of rTMS applied to the left DLPFC can have short-term therapeutic benefits for negative symptoms although studies have not yet tested treatment regimens capable of producing longer term/persistent benefits (Garg et al., 2016; Tseng et al., 2022).
**Obsessive-Convulsive Disorder (OCD)**
Deep TMS coil in the treatment of OCD involves the use of high-frequency stimulation using a custom H7 deep TMS coil (Carmi et al., 2019). Use of an angled figure of eight coil with a standard rTMS device has also been approved by the FDA and TGA for the treatment of OCD. rTMS approaches using standard figure of eight coils have targeted the bilateral supplementary motor cortex or left or right orbitofrontal cortex (OFC) with 1-Hz stimulation trains, although there is also (limited) evidence for DLPFC stimulation (Fitzsimmons et al., 2022; Perera et al., 2021). A recent meta-analysis showed that rTMS over the bilateral pre-SMA, the DLPFC (especially the bilateral DLPFC), the OFC (especially the right OFC), and the medial prefrontal (*mPFC*) and anterior cingulate (*ACC*) cortices (mPFC/ACC) seems to be more effective than sham stimulation in reducing OCD symptoms. The effect size ranges from small–moderate (TMS over the mPFC/ACC and OFC) to moderate (TMS over the pre-SMA and DLPFC). The moderator analyses did not show any clear clinical predictor of post-treatment Y-BOCS score reduction. The current evidence is still not sufficient to provide clear indications of a TMS protocol and/or a brain target over the others. This fact is at least partially due to the small sample size and heterogeneity of the TMS protocols and devices across studies (Grassi et al., 2023).
**Post-Traumatic Stress Disorder (PTSD)**
High- as well as low-frequency rTMS of the right DLPFC appears to significantly reduce core PTSD symptoms in patients with PTSD. rTMS may therefore be a promising alternative or add-on treatment for PTSD patients who show limited response to antidepressant medication and/or trauma-focused psychotherapy. More high-quality studies are necessary to explore the effects on different symptom clusters in PTSD (Kan et al., 2020).
**Addiction**
Excitatory rTMS of the left dorsolateral prefrontal cortex (DLPFC) significantly reduced craving compared with sham stimulation. Moreover, meta-regression revealed a significant positive association between the total number of stimulation pulses and effect size among studies using excitatory left DLPFC stimulation. Effects of other rTMS protocols on craving were not significant. However, when examining substance consumption, excitatory rTMS of the left DLPFC and excitatory deep TMS (dTMS) of the bilateral DLPFC and insula revealed significant consumption-reducing effects, compared with sham stimulation. The anti-craving effect may be associated with stimulation dose (Zhang et al., 2019). Studies suggest that rTMS applied to DLPFC is a safe and promising therapeutic technique for the management of comorbid schizophrenia and substance use disorders (Johnstone et al., 2022). Studies into smoking abstinence found that the response rate was higher when excitatory rTMS was used on the left DLPFC or when using deep rTMS targeting the lateral prefrontal cortex and insula bilaterally. It was also determined, using grades of recommendation, assessment, development and evaluation, that overall there was a low level of confidence in the results (Petit et al., 2022).
**Chronic pain**
Overall, findings support that HF-DLPFC stimulation is able to induce an analgesic effect in chronic pain and in response to provoked pain (Che et al., 2021). Benefits have been seen in studies applying stimulation to the primary motor cortex (M1), to the DLPFC and using deep TMS. The most consistent promising effects seen in clinical trials have been present when stimulation has been applied to the left M1 although it has been proposed that optimal treatment should involve a systematic evaluation of multiple treatment targets in an individual patient (Lefaucheur and Nguyen, 2019).
**Post-stroke depression and cognition**
Overall, both HF-rTMS and LF-rTMS have been shown to be safe and well-tolerated (Fisicaro et al., 2019).

Some clinicians choose to switch to another form of rTMS if there has been no response after 15-20 treatments. However the evidence base does not currently show better clinical outcomes between switching from one protocol to another after 15 or 20 treatments versus continuing for a full 30 treatment sessions on the initial protocol.

## Potential side effects

Research to date indicates that rTMS is well-tolerated and safe when patients are carefully screened and treatment is given within recommended safety parameters and evidence-based guidelines. There are minimal risks with rTMS and any side effects are usually mild, transient and/or can be easily managed. The most commonly reported side effects with rTMS include local scalp pain or discomfort, headache, fatigue and facial muscle twitching during stimulation. The rate of clinical trial discontinuation due to adverse effects across 93 randomised controlled trials was 2.5% in patients receiving active, compared to 2.7% in patients receiving sham stimulation, supporting an excellent safety profile (Zis et al., 2020).

Clinical trials have found no cognitive impairment when rTMS is given within recommended parameters. On the contrary, improvement in cognitive function may be expected in patients whose depression respond to rTMS, particularly if cognitive impairment is a feature of their depressive syndrome.

More serious side effects (including risk of seizure and inducing a manic or hypomanic episode) are rare and these risks diminish when safety precautions are followed (Taylor et al., 2018). Monitoring and assessment prior to and during the treatment and treatment course, in line with these guidelines, will help to minimise risks. Psychiatrists should be aware of the contra-indications by familiarising themselves with guidelines relevant to safety:

- The incidence of induction of a generalised seizure has not been fully quantified but appears to be extremely low when patients are adequately screened for risk factors and treatment applied carefully (Rossi et al., 2021). An increased risk seems likely in patients with pre-existing neurological conditions, alcohol or substance use and possibly during changes in medications (particularly those that lower seizure threshold) during the rTMS course. All services offering rTMS are required to have protocols to manage seizure induction. Prompt cessation of rTMS is indicated in these instances. There is no evidence to suggest rTMS increases an individual’s risk of experiencing a seizure in the future.
- The other rare, but potentially serious adverse effect is that of inducing a manic or hypomanic episode. These episodes can occur most commonly in patients with a pre-existing diagnosis of bipolar affective disorder. In clinical experience they are rare in patients receiving mood-stabilising medication while undertaking rTMS.

Accelerated TMS (aTMS) studies had similar seizure and side effect incidence rates to those reported for once daily rTMS. One seizure was reported from aTMS (0.0023% of aTMS sessions, compared with 0.0075% in once daily rTMS). The most common side effects were acute headache (28.4%), fatigue (8.6%) and scalp discomfort (8.3%), with all others under 5% (Caulfield et al., 2022a, 2022b).

In general, tolerability of rTMS improves over the course of treatment and may be eased with simple analgesia such as paracetamol.

## Assessment prior to procedure

No specific pre-treatment preparation is required prior to a treatment session. Patients sit in a comfortable chair during the treatment sessions. Ear plugs or other hearing protection should be provided to minimise potential discomfort caused by noise generated by the coil.

Psychiatrists must ensure that a pre-rTMS evaluation is undertaken that includes a full psychiatric assessment, as well as consideration of relevant investigations if indicated. A medication review must also be completed prior to the administration of rTMS. The use of a structured safety screen is highly recommended (Keel et al., 2001; Taylor et al., 2018).

Before prescription of rTMS, patients should be assessed for factors which place them at greater risk of rTMS-related complications, especially seizures.

rTMS is associated with a small risk of treatment-emergent affective switching. This should be discussed with all patients, particularly those with a history of bipolar affective disorder in whom the risk may be increased. The use of mood-stabilising medication in patients with bipolar affective disorder, particularly those with a history of manic switching, would seem likely to reduce this risk but has not been formally evaluated.

### Monitoring during treatment sessions and the treatment course

During each course of therapy, patients should be monitored by appropriately trained and supervised clinicians^2^ including ongoing assessment of mental state, treatment response and any side effects should be reviewed as well as any unusual experiences.

Appropriate facilities to manage any complications from rTMS, including seizures, should be available.

Daily monitoring of patients by clinicians should include assessment of factors that may alter the seizure threshold. This includes any changes in medication prescribed, alcohol or other substance use, and evidence of acute neurological symptoms or a decline in physical health.

Protocols should be in place to allow for the timely management of the common side-effects of rTMS including scalp discomfort and headache. Pain can improve over the course of the treatment, and headache may be eased with the use of analgesia. A switch from high-frequency to low-frequency stimulation protocols may be warranted if these side-effects continue to be a barrier to treatment continuation.

Clinicians supervising rTMS therapy should have the capacity to identify signs of an emergent manic switch and have protocols in place to respond appropriately.

Tapering of rTMS sessions may be appropriate at the end of an acute course of therapy: this is a process where treatment frequency is gradually reduced from five days per week to a lesser frequency (O’Reardon et al., 2007). This should be differentiated from ongoing maintenance rTMS.

Ongoing pharmacotherapy is recommended to prevent relapse after an acute course of rTMS and this practice is reflected in research studies, including the seminal trial by O’Reardon et al., 2007.

## rTMS use with other treatments

rTMS should be considered as part of the spectrum of treatment options currently available. Treatment with rTMS can occur in combination with psychological therapies or medications. Studies confirm this depends on the care needs and symptom profile of the individual patient.

The current state of understanding of rTMS and ECT indicate these treatment modalities have distinct mechanisms of action and side effect profiles, and therefore are best considered distinct therapeutic modalities in their own right. Patients with depression who have not responded to one modality may gain useful clinical benefit from the other treatment. Only very limited research has explored the concurrent combination of both treatments to date, and therefore the use of ECT and rTMS concurrently should occur only within a research protocol approved by a local ethics committee.

## Maintenance rTMS

Maintenance rTMS is the provision of regular treatment sessions to patients who are in remission or who have minimal ongoing depressive symptoms, in order to prevent depressive relapse.

There is emerging evidence that maintenance rTMS strategies can be used to prevent relapse but further research is required to define the most effective and efficient strategies (d’Andrea et al., 2023; Fitzgerald, 2019). The use of maintenance rTMS should be considered in the broader context of other relapse prevention strategies. Given the limited evidence base currently, the clearest indication for maintenance rTMS is in those patients who have responded well to TMS but experienced a relatively early return of symptoms – patients would receive a second course of treatment to restore remission followed by maintenance to try and prolong clinical benefits. The history of relapses prior to maintenance rTMS could be compared with history of relapse post-maintenance rTMS to determine if maintenance TMS has been beneficial (Fitzgerald, 2019). Maintenance rTMS should also be considered in patients with clinical characteristics associated with a high risk of relapse after acute treatment, including those with high treatment resistance.

Two main approaches to maintenance rTMS have been evaluated in the literature or tried in clinical practice. In the first, single rTMS sessions are applied at a frequency that might vary between two per week to one per month depending on clinical need. In the second approach, a cluster of four – six treatments are provided over several days at a frequency of once every 3 to 6 weeks (Pridmore et al., 2018; Wang et al., 2017).

## rTMS retreatment

Further acute courses of rTMS may be offered to patients who have experienced a partial or full relapse of depressive symptoms. There is relatively limited literature to date that has explored the reintroduction of rTMS in patients experiencing a relapse but this literature suggests that patients who have initially responded to rTMS are highly likely to do so with subsequent treatment (Demirtas-Tatlidede et al., 2008; Dunner et al., 2014; Fitzgerald et al., 2006; Janicak et al., 2010; Kelly et al., 2016; Philip et al., 2016; Pridmore et al., 2019).

Repeat courses are appropriate for patients who have had a demonstrated positive response to the initial course, and there is no clinical reason to restrict TMS to a maximum number over a person’s lifetime, given the excellent safety profile and as relapse can happen at any time. The number of sessions required may be given flexibly according to the patient’s individual needs.

The frequency of retreatment would again vary depending on the individual needs of the patient. Across the majority of studies, the time to repeat treatment averaged between 4 and 10 months. It is important to note however that the duration until reintroduction of treatment was fairly consistently dependent on the degree of initial response achieved by patients (Dunner et al., 2014). In other words, patients who achieved a more complete remission in their original index course of treatment seem to remain well for longer periods of time.

## Privacy and professional practice issues

Administration of rTMS should be conducted in a respectful manner and privacy should be maintained throughout the procedure. It is not appropriate for a patient to be receiving rTMS when another patient is waiting for treatment in the same room or having two patients being treated in the same space without some form of isolating barrier in place.

## Skills required for delivering rTMS

### rTMS by psychiatrists

All psychiatrists who are administering rTMS should be credentialed by their institution for rTMS treatment. Every service offering rTMS should have a process for the assessment and subsequent credentialing and re-credentialing of psychiatrists who administer rTMS to ensure that they meet required professional standards. This should be undertaken and monitored in accordance with local governance systems. Institutions that deliver rTMS should detail their credentialing requirements in a local policy document.

The capacity to undertake rTMS management should take into consideration:

1. Competence in performing assessments of suitability to undergo rTMS and ability to conduct rTMS treatments across a range of clinical situations
2. Demonstration of maintenance of knowledge and practical skills through continuing education and practice improvement activities in rTMS (including recognised rTMS courses, conferences, peer review groups, quality improvement activities).
3. Appropriate training for psychiatrists is likely to require participation in a relevant course that features a certification process. Details of courses that have been endorsed by the RANZCP, as well as information on the expected standards and criteria for these courses can be found on the RANZCP rTMS course endorsement website. The RANZCP has noted the following guidelines and adapted these to the Australian context: Training in the practice of non-invasive brain stimulation: Recommendations from an International Federation of Clinical Neurophysiology (IFCN) committee (March 2021).
4. Basic practical knowledge from a device manufacturer is unlikely to provide adequate depth and breadth of knowledge needed for clinical applications of rTMS.

To be credentialed to prescribe or administer rTMS, a formal assessment of the psychiatrist’s practical skills in rTMS administration should also be conducted by a site director or equivalent, e.g. an rTMS-credentialled psychiatrist. The determination is then made that the required standard has been met as a means of practice review and quality assurance. The psychiatrist performing the credentialing should have expertise and detailed knowledge of current rTMS practice.

### rTMS by psychiatry trainees or other health care professionals

When rTMS is administered as a treatment for psychiatric disorders by a psychiatry trainee or other clinician (e.g. psychiatric nurse), this should be done under the supervision of a psychiatrist who has professional training in rTMS and is credentialed as detailed above.

### Ongoing education

It is acknowledged that rTMS is a specialised and evolving practice. It is important to ensure clinical and technical application is carried out optimally for each individual patient. There should be continuing professional education to ensure all clinicians involved in the provision of rTMS treatment keep up to date on clinical indications and treatment advances.

Psychiatrists working in this field will need to ensure they meet the following ongoing CPD requirements for rTMS, as outlined by the RANZCP:

- reference of rTMS in their Professional Development Plan
- a minimum of 10 hours of CPD activities completed annually relating to rTMS. These hours can be spread across any of the following CPD sections:
  - Section 2: Formal Peer Activities (e.g., participation in an rTMS Peer Review group)
  - Section 3: Practice Improvement Activities (e.g. audit of an rTMS practice or service).
  - Section 4: Self-Guided Learning Activities (e.g. attending rTMS workshops, neurostimulation conferences and reading journal articles).

Where required, psychiatrists may need to engage in peer review or primary/secondary consultation processes to determine the appropriateness of rTMS for a given patient. Collaboration, peer review and sharing of knowledge and experience across psychiatrists practising rTMS are recommended.

## Outcome-based measures

It is essential for all services delivering rTMS to have systems in place for monitoring of efficacy, outcomes and treatment-related adverse effects of treatment. Clinical and psychometric assessment of symptom severity before, during and at the end of treatment is highly recommended. These measures should be incorporated into routine clinical practice to guide treatment planning. A regular clinical audit process, conducted at least annually, should also be in place to ensure high quality, patient-focused treatment is always delivered.

## Governance

Each clinical setting that conducts rTMS treatment should have in place formal policies and procedures which govern

- the clinical assessment of patients considered for rTMS and its prescription, incorporating evidence-based stimulation parameters and consideration of appropriate clinical indications;
- the qualifications, training and credentialing of clinicians involved in rTMS provision;
- the process for monitoring outcomes, including both efficacy outcomes and adverse events;
- maintenance and servicing of rTMS machines and ancillary equipment.

Each service that conducts clinical rTMS treatment should have a process for ensuring adequate training of clinicians delivering rTMS and a process of credentialing, such that practitioners have appropriate levels of both theoretical knowledge and practical experience. All clinicians who administer rTMS should be properly trained in the theory, technique and safe operation of rTMS. Each service should have a formal time period for re-credentialing of personnel involved with rTMS.

## Research

Psychiatrists should contribute to continued service development, quality improvement and research by monitoring treatment outcomes. This is important for both established and evolving rTMS techniques to contribute to a more complete understanding and improvement in clinical practice.

Further optimisation of treatment protocols, and efficacy in different patient groups, and other psychiatric conditions are important foci of ongoing research.

## Conclusion

These guidelines provide up-to-date information for psychiatrists to enhance their commitment to promoting optimal standards of rTMS practice. The guidelines should be an integral feature of training and professional development activities and considered by rTMS service providers. The RANZCP hopes that these guidelines will contribute substantially to assist psychiatrists in attaining a high standard of professional practice which will benefit their patients. These guidelines will be reviewed every 3 years to maintain currency and usefulness to practice.

## Notes

1. The term patient is used through this document for clarity and consistency although it is recognised that individuals may prefer alternative terms, for example person with lived experience, consumer, client or service user.

2. Clinicians in the context of these guidelines include psychiatrists or appropriately trained healthcare professionals, such as a psychiatric nurse, who are administering rTMS or involved in delivering the rTMS treatment under the supervision of a psychiatrist.
